# Anaemia among Pregnant Women Visiting Obstetric Department of a Tertiary Care Centre: A Descriptive Cross-sectional Study

**DOI:** 10.31729/jnma.7967

**Published:** 2023-01-31

**Authors:** Padam Raj Joshi, Saroj GC, Sushil Sah, Reshika Shrestha, Niharika Pathak, Sujata Maharjan, Pooja Paudyal

**Affiliations:** 1 Maharajgunj Medical Campus, Maharajgunj, Kathmandu, Nepal; 2 Bajrabarahi Chapagaun Hospital, Bajrabarani, Lalitpur, Nepal; 3 Department of Gynaecology and Obstetrics, Maharajgunj Medical Campus, Tribhuvan University Teaching Hospital, Maharajgunj, Kathmandu, Nepal

**Keywords:** *anaemia*, *pregnant women*, *prenatal care*, *tertiary care centre*

## Abstract

**Introduction:**

Anaemia is one of the common health problems of pregnant women and children in developing countries. Anaemia in pregnancy is related to poor foetal and maternal outcomes contributing to significant morbidity and mortality. Anaemia is a treatable and preventable condition. The objective of this study was to find out the prevalence of anaemia in pregnant women visiting the Obstetric Department of a tertiary care centre.

**Methods:**

A descriptive cross-sectional study was conducted among the pregnant women visiting the Department of Obstetrics and Gynecology of a tertiary care centre for their antenatal checkup. The study was conducted from 2 November 2022 to 11 November 2022 after obtaining ethical approval from the Institutional Review Committee [Reference number: 11(6-11)E2/079/080]. Pregnant women with a history of blood transfusion, anaemia of chronic disease like chronic kidney disease, history of recurrent bleeding, and referral cases from other centres were excluded from the study. Serum haemoglobin was used for diagnosing anaemia according to criteria given by the World Health Organization. Convenience sampling was used. Point estimate and 95% Confidence Interval were calculated.

**Results:**

Among 442 pregnant women, the prevalence of anaemia was 24 (5.43%) (3.32-7.54, 95% Confidence Interval).

**Conclusions:**

The prevalence of anaemia among pregnant women was lower in comparison to other studies done in similar settings.

## INTRODUCTION

Anaemia is one of the global public health problems, with a global prevalence of 29.90% in women of reproductive age and 36.50% in pregnant women.^1^ Overall prevalence of anaemia among women of reproductive age in Nepal is 43%.^2^ The main contributing factor to anaemia during pregnancy in developing countries is nutrition.^3^ Among nutritional anaemia iron deficiency anaemia is the most common cause.^4^

Anaemia during pregnancy is associated with poor maternal and child health.^5^ It is easier to diagnose anaemia during pregnancy, is of low cost and screening can be applied even in rural settings. Moreover, anaemia is preventable and treatable. Treating anaemia early can prevent these foetal and maternal complications.^1,3,5^

The aim of this study was to find out the prevalence of anaemia in pregnant women visiting the Obstetric Department of a tertiary care centre for an antenatal care (ANC) checkup.

## METHODS

A descriptive cross-sectional study was conducted among pregnant women visiting for their antenatal checkup in the Department of Obstetrics and Gynecology of Tribhuvan University Teaching Hospital, Kathmandu, Nepal. The data was collected from 2 November 2022 to 11 November 2022 after obtaining ethical approval from the Institutional Review Committee of the same institute [Reference number: 11 (6-11 )E^2^ 079/080]. All pregnant women visiting within the study period were included in the study. Pregnant women with a history of blood transfusion, anaemia of chronic disease like CKD, history of recurrent bleeding, and referral cases from other centres were excluded from the study. Convenience sampling method was used. The sample size was calculated using the following formula:


$$\begin{array}{ll} \text{n} & ={\text{Z}}^{2}\times \frac{\text{p}\times \text{q}}{{\text{e}}^{\text{2}}}\\ & =1.96^{2}\times \frac{0.622\times 0.378}{0.05^{2}}\\ & =362 \end{array}$$

Where,

n = minimum required sample size
Z = 1.96 at 95% Confidence Interval (CI)
p = prevalence of anaemia among pregnant women taken as 62.2%^6^
q = 1-p
e = margin of error, 5%

The calculated minimum required sample size was 362. However, a total of 422 pregnant women were included in the study.

Data was collected from an ANC card using the predesigned proforma. Already recorded haemoglobin level and other routine blood investigations, demographic details, height, weight, gravidity, parity, abortion, age at first pregnancy, interpregnancy interval, obstetrics, and medical and surgical history were noted. Serum haemoglobin was used for diagnosing anaemia as per World Health Organisation (WHO) guidelines. According to WHO, haemoglobin levels of less than 11 g/dL were diagnosed as anaemia in pregnant women. The different haemoglobin levels for each anaemia class were 10.0-10.9 g/dL for mild, 7-9.9 g/dL for moderate and less than 7 g/dL for severe anaemia.^3^

The collected data were entered in Microsoft Excel 2016. The completeness of data was ensured by another investigator and duplication was removed. Data were analysed using Microsoft Excel 2016 and IBM SPSS Statistics version 18.0. Point estimate and 95% CI were calculated.

## RESULTS

Out of 442 pregnant women, anaemia was found in 24 (5.43%) (3.32-7.54, 95% CI). There were 15 (62.50%) who had mild anaemia, 9 (37.50%) with moderate anaemia and no severe anaemia (Table 1).

**Table 1:** The severity of anaemia (n = 24).

Severity of anaemia	n (%)
Mild	15 (62.50)
Moderate	9 (37.50)
Severe	-

A total of 18 (75%) anaemic pregnant women were in the age group of 20-30 years. Homemakers were 15 (62.50%) by occupation (Table 2).

**Table 2:** Demographic profile of anaemic patients (n = 24).

Characteristics		n (%)
Age (years)	15-19	1 (4.17)
	20-24	9 (37.5)
	25-29	9 (37.5)
	30-34	4 (16.67)
	35-40	1 (4.17)
Occupation	Homemaker	15 (62.50)
	Student	3 (12.50)
	Business	3 (12.50)
	Others	3 (12.50)
Education	Primary	10 (41.67)
	Secondary	10 (41.67)
	Bachelor	3 (12.50)
	Master	1 (4.16)

Most of the women 17 (70.83%) had zero parity. There were 12 (50%) women who were overweight and obese. The most common comorbidity was rheumatic heart disease, contributing to about 3 (12.50%) ofanaemic women (Table 3).

**Table 3:** Clinical profile of anaemic patient (n= 24).

Characteristics		n (%)
Parity	0	17 (70.83)
	1	4 (16.67)
	2	3 (12.5)
Abortion	0	17 (70.83)
	1	6 (25)
	>1	1 (4)
Gravida	1	13 (54.17)
	2	5 (20.83)
	>2	6 (24.9)
Age at first pregnancy	15-19	2 (8.33)
	20-24	12 (50)
	25-29	8 (33.33)
	30-34	2 (8.33)
BMI	18.5-22.9	12 (50)
	23-24.9	6 (25)
	>25	6 (25)
Coexisting disease conditions	RHD	3 (12.50)
	Thrombocytopenia	3 (12.50)
	Hypothyroidism	2 (8.33)
	Hypertension	1 (4.17)

## DISCUSSION

The prevalence anaemia in pregnant women in our study was 5.43% which was low in comparison to the prevalence of 62.2% derived from a study done in 1995 in Kathmandu valley showing a decreasing trend of anaemia in our setting.^6^ A study done in 2018 in the Karnali province of Nepal has shown an anaemia prevalence of 17.9% which is almost 3 times higher than that in our setting.^7^ A study was done at the village development committee level in 2014 with a reported prevalence of anaemia as 46.6% which is higher in comparison to our setting.^8^ Overall worldwide prevalence of anaemia in pregnant ladies derived from systematic review and meta-analysis were done in 2022 is 36.8%.^9^ WHO has reported a prevalence of 40%.^10^ Both of these worldwide reported prevalence is higher in comparison to our setting. There is also a decreasing trend of anaemia during pregnancy in many low and middle-income countries similar to our setting.^11^ In our study anaemia was found to be more prevalent in prime gravida than in successive pregnancies. Whether this decline in the prevalence of anaemia in successive pregnancies is due to effective screening needs to be established by further study in future.

Though the prevalence of anaemia was found to be low in our setting compared to other similar studies, timely treatment of this helps to improve maternal and foetal outcomes. Systematic review and metaanalysis have established that anaemia during the first trimester is associated with preterm birth, low birth weight and increased NICU admission resulting in a poor foetal outcome. Along with poor fetal outcomes anaemia also increases maternal complications like pre-eclampsia, antepartum haemorrhage, postpartum haemorrhage and also increased number of cesarean deliveries.^3,5,12,13^ Low haemoglobin level during the first trimester is also associated with an increased risk of first-trimester miscarriage.^14^

The main contributing factor for anaemia during pregnancy in developing countries is nutritional.^3^ Among nutritional anaemia iron deficiency anaemia is the most common cause.^4^ It is easier to diagnose anaemia during pregnancy, is of low cost and screening can be applied even in rural settings as well. Moreover, Anaemia is preventable and treatable. Treating early in the first trimester can prevent all these foetal and maternal complications. So the screening program during the first trimester is very important for early treatment.

The prevalence determined in this study is done in a single centre with a convenient sampling method in a short duration. So, this may not reflect the overall burden of anaemia during pregnancy in a larger population.

## CONCLUSIONS

The prevalence of anaemia among pregnant women was found to be low in comparison to other studies done in a similar setting. But, this much-reported anaemia affects maternal and foetal health signifying the importance of screening and treatment of anaemia during pregnancy. So, the concerned stakeholders should consider the prevention of anaemia as a public health intervention.
